# Legionnaires’ Disease Mortality in Guinea Pigs Involves the p45 Mobile Genomic Element

**DOI:** 10.1093/infdis/jiz340

**Published:** 2019-07-02

**Authors:** Lanette M Christensen, Preeti Sule, Suat L G Cirillo, Madison Strain, Quinci Plumlee, L Garry Adams, Jeffrey D Cirillo

**Affiliations:** 1 Department of Microbial Pathogenesis and Immunology, College of Medicine, Texas A&M University Health Science Center, Bryan; 2 Department of Veterinary Pathobiology, Texas A&M University, College Station

**Keywords:** pathogenesis models, infection, inflammatory response, interferon gamma, *Legionella pneumophila*

## Abstract

**Background:**

*Legionella* can cause Legionnaires’ disease, a potentially fatal form of pneumonia that occurs as sporadic epidemics. Not all strains display the same propensity to cause disease in humans. Because *Legionella pneumophila* serogroup 1 is responsible for >85% of infections, the majority of studies have examined this serogroup, but there are 3 commonly used laboratory strains: *L pneumophil*a serogroup 1 Philadelphia (Phil-1)-derived strains JR32 and Lp01 and 130b-derived strain AA100.

**Methods:**

We evaluated the ability of Phil-1, JR32, Lp01, and AA100 to cause disease in guinea pigs.

**Results:**

We found that, although Phil-1, JR32, and AA100 cause an acute pneumonia and death by 4 days postinfection (100%), strain Lp01 does not cause mortality (0%). We also noted that Lp01 lacks a mobile element, designated p45, whose presence correlates with virulence. Transfer of p45 into Lp01 results in recovery of the ability of this strain to cause mortality, leads to more pronounced disease, and correlates with increased interferon-γ levels in the lungs and spleens before death.

**Conclusions:**

These observations suggest a mechanism of Legionnaires’ disease pathogenesis due to the presence of type IVA secretion systems that cause higher mortality due to overinduction of a proinflammatory response in the host.

Legionnaires’ disease is a potentially fatal form of pneumonia that occurs primarily in individuals over the age of 55 years old, smokers, and those who suffer from cystic fibrosis [1, 2]. The causative agent, *Legionella pneumophila*, was originally identified after infections were produced by aerosol droplets from a contaminated air cooling tower in 1976 [2, 3]. The strain responsible was designated *L pneumophila* serogroup (SG)1 strain Philadelphia (Phil-1). Clinically relevant infections are characterized by severe peribronchiolar pneumonia leading to multiorgan failure and death in many cases [2]. Influx of neutrophils appears to be responsible for pneumonia and may be triggered by an inflammatory response mediated by nuclear factor (NF)-κβ and interleukin (IL)-1, possibly induced through inhibition of host cell protein synthesis by some of the over 300 *Legionella* effectors [4–8]. The neutrophils and monocytes in the infiltrate allow enhanced interferon (IFN)-γ, IL-12, and tumor necrosis factor (TNF) production that are both necessary for control of infection and create the Legionnaires’ pneumonia [4, 9, 10]. Factors that determine whether the host response is tipped toward overinduction and mortality or optimal balance and clearance are not well understood.

Over 85% of clinical cases of Legionnaires’ are due to SG1 strains of *L pneumophila* [11]. Overrepresentation of certain strains is not simply because disease-causing *L pneumophila* are more prevalent in the environment, rather they have acquired the ability to infect and persist within human hosts [11–14]. Presumably, strains that cause disease gained the necessary functions through coevolution with environmental hosts [15–18]. However, approximately 80% of the environmental *Legionella* have never been associated with clinical Legionnaires’ disease [11, 12]. These observations suggest that *Legionella* spp are predominately environmental inhabitants, making humans incidental hosts [1, 2, 19]. *Legionella* have the ability to withstand many environmental stressors including temperature [20, 21], osmotic pressure, pH, nutrient deprivation [19], and many antimicrobials, due to intricate regulation [22, 23] and genomic plasticity [24]. We found that *Legionella* strains associated with clinical infections carry specific loci, some of which are on mobile elements and temperature regulated [13, 21]. It has been observed that differences in secretion correlate with virulence [25], suggesting that research on secretion systems present in *Legionella* would provide insight into pathogenesis [26, 27]. It is interesting to note that, although the type IVB system, designated *dot*/*icm*, is present in all *Legionella*, the type IVA system, designated *lvh* and present on the mobile element p45, shows a greater prevalence in disease-causing strains [13, 21, 28–32]. Strains Lp01 and JR32 were separately derived from the parent strain SG1 strain Phil-1. Strain JR32 maintained the presence of p45, whereas strain Lp01 no longer carries p45 [13, 31–33]. The SG1 strain AA100, also designated by others as 130b or Wadsworth, carries p45 [13, 34] and is thought to have recently diverged from Phil-1 and other SG1 strains [31]. Based on these data, examination of the virulence of these strains might provide insight into the contribution of p45 to virulence and whether the correlation of p45 with disease is due to a role in virulence.

We compared the closely related SG1 strains Phil-1, AA100, JR32, and Lp01 for their ability to cause disease in guinea pigs after infection by aerosol. Lp01, lacking the p45 mobile genomic element (MGE), did not cause mortality in guinea pigs; however, 100% of the guinea pigs infected by the other strains succumbed to infection. Contribution of p45 to this difference in pathogenesis was examined by introduction of p45 into Lp01 from Phil-1. The pathogenesis of the resulting strain, Lp01p45kan, was compared with Lp01 and Phil-1 in guinea pigs and restores the ability to cause mortality. We observed that pathology in the lungs as well as TNF-α, IFN-γ, and IL-12 levels correlate with p45, suggesting that in *L pneumophila* p45 plays a role in severity of Legionnaires’ disease through a mechanism involving overinduction of a proinflammatory cascade.

## MATERIALS AND METHODS

### Bacterial Strains and Growth Conditions

*Legionella* strains were prepared and grown as described in detail in the Supplementary Information.

### Confirmation of Chromosomal Integration and Presence of p45

The presence of episomal and replicating forms of p45 were confirmed as described in the Supplementary Information.

### Vector Construction

Vectors were constructed as described in the Supplementary Information.

### Conjugation of p45

The p45 element was moved between strains by conjugation as described in the Supplementary Information.

### Confirmation of Lp01^p45kan^

Movement of p45 and verification of the integrity of the strain containing it was conducted as described in the Supplementary Information.

### Transcript Analysis in *Legionella*

Analysis of transcription in *Legionella* was carried out as described in the Supplementary Information.

### Sequencing of *Legionella* Genomic Regions

Sequences of relevant genomic regions in the *Legionella* strains were determined as described in the Supplementary Information.

### Guinea Pig Infections

Hartley guinea pigs were infected with *Legionella* by aerosol as described in the Supplementary Information.

### Cytokine Expression Analyses

Expression levels of cytokines in guinea pigs were measured as described in the Supplementary Information.

### Histopathology

Histopathology was done on guinea pig tissues as described in the Supplementary Information.

### Ethics Statement

All animal experiments performed in this study were approved by the Institutional Animal Care and Use Committee at Texas A&M University (no. IACUC-2017-0361) in compliance with National Institutes of Health guidelines as described in the Guide for the Care and Use of Laboratory Animals.

### Statistical Analysis

The significance of the results was determined by analysis of variance (ANOVA). When significant variance was determined between groups by ANOVA, the Student’s *t* between-means test was used. *P* values less than .05 were considered significant. Microsoft Excel and GraphPad Prism software were used for statistical analysis and production of figures.

## RESULTS

### Common *Legionella* Strains Differ in Virulence

**Figure 1.**
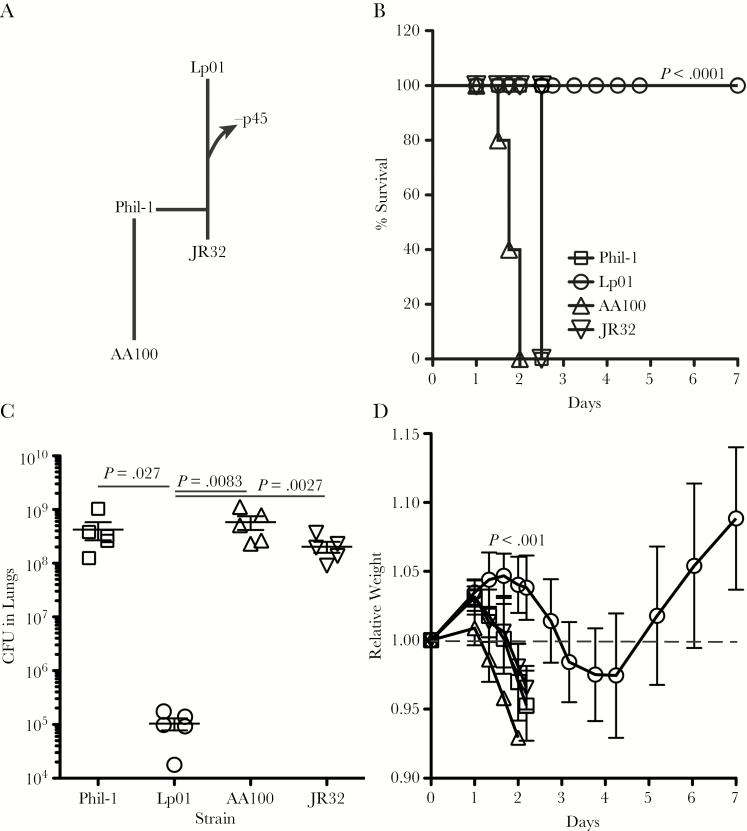
Common laboratory strains differ in virulence for guinea pigs. Relationship between the 4 strains used in this study (A). AA100 is thought to have recently diverged from other *Legionella pneumophila* serogroup 1 strains, including Philadelphia (Phil-1). Both JR32 and Lp01 are directly derived from Phil-1, but only Lp01 lost p45 during this process, whereas JR32 still carries p45. Percentage of survival after infection of 5 guinea pigs with Phil-1 and its derivatives Lp01 and JR32 and 130b strain AA100 by aerosol and bacterial burden in the lungs at the time of death (B). Error bars in B and C are standard deviations, and horizontal lines in B indicate mean values for the group. *P* values shown are from the log-rank test (B), Student’s *t* test (C), or analysis of variance (D). All statistical comparisons are between other strains and Lp01. Figure legend in B applies to B–D. Relative weight is calculated as the weight of each guinea pig at each time point divided by their weight at day zero. CFU, colony-forming units.

*Legionella pneumophil*a serogroup 1 Philadelphia is the original isolate from which JR32 and Lp01 were separately derived (Figure 1A) [13, 31–33]. Sequencing and analysis of Lp01 found that it lacks p45 [32], as shown by our earlier analyses [13]. Although it has been reported that there are specific mutations in Lp01 isolates [32], it was unclear whether our Lp01 isolate carried the same mutations. We sequenced the regions previously found to differ between Phil-1 and Lp01 [31], and, other than the confirmed loss of p45 by Lp01, only the 9-base pair (bp) deletion in *ndh* and point mutations in *rpsL* and *luxN* are present in our Lp01 isolate (Supplementary Table S2). The point mutations described by Chien et al [31] to be present within lpg0716, lpg0718, and *ftsE* in Lp01 are not present in our isolate, increasing the likelihood that phenotypic characteristics of our Lp01 are due to loss of p45. Sequences we obtained for Phil-1 match that reported [31, 32], and a summary of our sequencing is provided in Supplementary Table S2. Because the primary difference between Lp01 and Phil-1 is the presence of p45 and strains AA100 and JR32 also carry p45 [13, 31–33], comparison of the virulence of these strains can provide insight into the role of p45 in disease. Guinea pigs were infected with Phil-1, Lp01, AA10, and JR32 via aerosol (Figure 1B). Strains Phil-1, JR32, and AA100 caused 100% mortality in guinea pigs within 3–4 days postinfection, but all Lp01-infected guinea pigs survived. This difference in virulence correlated with a 3- to 4-log difference in bacterial load within the lungs at the time of death for Phil-1, JR32, and AA100 or 7 days postinfection for Lp01 (Figure 1C). Guinea pigs infected with Phil-1, JR32, and AA100 rapidly progressed to mortality, with increasing signs of respiratory distress after 24 hours and continued weight loss before humane euthanasia to prevent suffering (Figure 1D). In contrast, Lp01-infected guinea pigs gained weight for 2 to 3 days before going through a relatively minor illness as observed by slight weight loss (<5%), followed by apparent full recovery and weight gain. These observations suggest that differences between Lp01 and Phil-1, JR32 and AA100 impact the ability of *L pneumophila* to cause mortality.

### Conjugation of the p45 Element Into Lp01 From *Legionella pneumophil*a Serogroup 1 Philadelphia 

**Figure 2.**
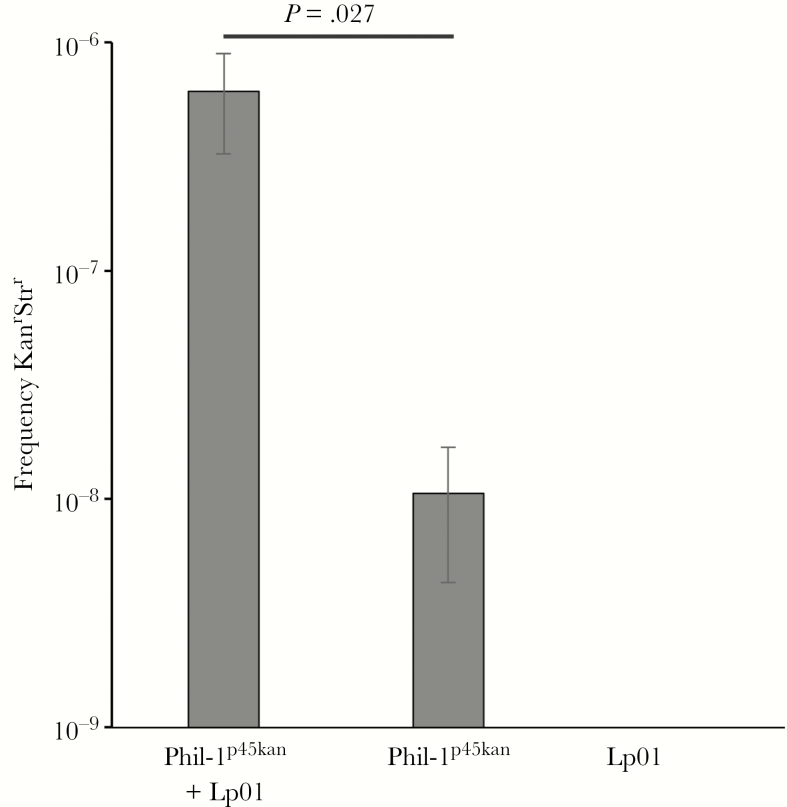
Conjugation of p45 into Lp01. Transfer of p45 into Lp01 by conjugation was confirmed by comparing the frequency of double kanamycin and streptomycin resistance observed when both *Legionella pneumophila* serogroup 1 Philadelphia wild-type strain (Phil-1) carrying p45^kan^ (Phil-1^p45kan^) and Lp01 are mixed compared with Phil-1^p45kan^ or Lp01 alone. Phil-1^p45kan^ mixed with an equal number of colony-forming units of Lp01, strain Phil-1^p45kan^ alone, or strain Lp01 alone were plated onto kanamycin and streptomycin containing plates to determine the frequency of double resistance in each population of bacteria. Frequency of kanamycin and streptomycin resistance (Kan^r^ Str^r^) was determined by dividing the number of colonies obtained on kanamycin and streptomycin containing plates by the number of colony-forming units placed on the plate. Data shown are mean values for triplicate samples and error bars represent standard deviations. The *P* value shown was obtained using the Student’s *t* test between the mixture of Phil-1^p45kan^ and Lp01 compared with Phil-1^p45kan^ alone.

Because p45 is likely responsible for the difference in virulence between Lp01 and Phil-1, we attempted to complement the virulence defect with p45. We marked p45 with kanamycin resistance (*aph*) to allow selection in *Legionella* by homologous recombination between the *aph*-containing plasmid pJDC305 and p45 in Phil-1 using a 200-bp region within the *lpg1237* gene on p45 that encodes a putative type II restriction enzyme. Recombination between pJDC305 and p45, producing p45kan, was confirmed by polymerase chain reaction (PCR). Because p45 is thought to encode conjugation machinery [26, 31], the resulting strain Phil-1^p45kan^ was used to conjugate p45kan into Lp01. Lp01 is streptomycin resistant, allowing selection for transfer with streptomycin and kanamycin double antibiotic selection. We confirmed transfer by comparing the frequencies of double streptomycin and kanamycin resistance in Phil-1^p45kan^ and Lp01 mixed conjugations with the frequency of Phil-1^p45kan^ or Lp01 alone (Figure 2). We obtained almost 100-fold higher frequencies of double resistance in mixed conjugations compared with either strain alone and no double-resistant colonies with Lp01 alone. These data suggest that p45kan can be successfully conjugated into Lp01, producing Lp01^p45kan^, at a frequency of approximately 1 in 10^6^ Phil-1^p45kan^ donor bacteria.

### Validation of p45 Transfer

**Figure 3.**
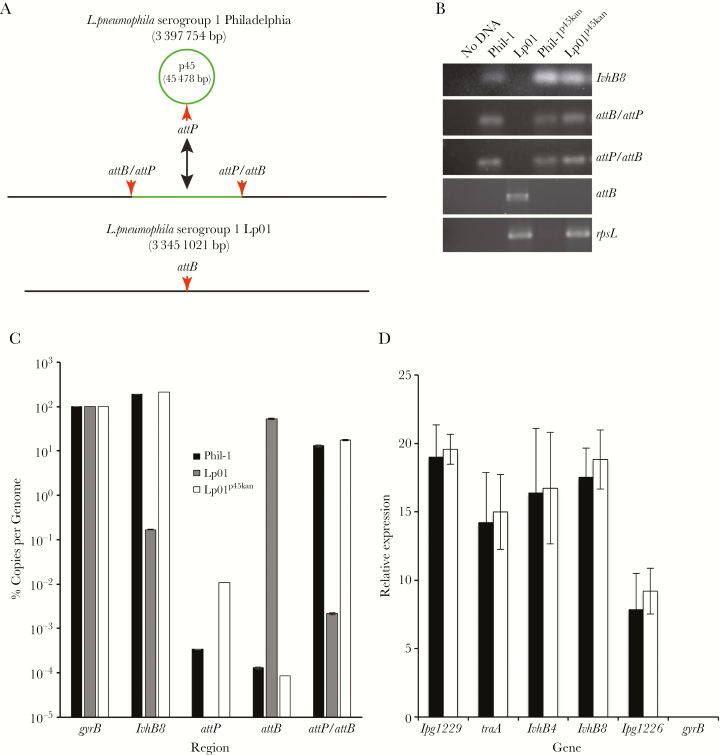
Confirmation and validation of p45 transfer into Lp01. Graphic representation of integrated and episomal forms of p45 present in *Legionella pneumophila* serogroup 1 Philadelphia wild-type strain (Phil-1) and the Lp01 chromosomal region (A). The size in base pairs of each deoxyribonucleic acid (DNA) molecule are shown in parentheses, site-specific recombination *attB*, *attP*, and the hybrid *attP*/*attB* or *attB*/*attP* sites that result from site-specific recombination are indicated by red arrows, and p45 is shown in green. Confirmation of relevant regions within strains Phil-1, Lp01, Phil-1^p45kan^, and Lp01^p45kan^ by polymerase chain reaction ([PCR] B). Total DNA was isolated from each strain indicated on the top of each column, and the PCR target region is shown to the right of each row. No DNA indicates the no-template control under the same PCR conditions as the total DNA templates from each strain. Quantitative PCR using total DNA from Phil-1, Lp01, and Lp01^p45kan^ to determine the number of copies of each region of DNA per genome calculated as the percentage of *gyrB* regions present (C). Expression of transcripts present on p45 in Phil-1 and Lp01^p45kan^ as measured by quantitative reverse-transcriptase PCR relative to *gyrB* transcripts in Lp01 using the 2^−ΔΔC^T method (D). Figure legend in C applies to C and D. Data are representative of 3 independent experiments in triplicate. Data points and error bars indicate means and standard deviations, respectively.

Strains Phil-1, Phil-1^p45kan^, Lp01, and Lp01^p45kan^ were evaluated by PCR for the presence of integrated and episomal forms of p45 (Figure 3A–C). All strains carry the expected regions, including the presence of p45 in Phil-1, Phil-1^p45kan^, and Lp01^p45kan^, the presence of attB in only Lp01, a streptomycin resistance mutation in *rpsL* only in Lp01 and Lp01^p45kan^, and *attB/attP* and *attP/attB* hybrid sites in strains that carry p45 (Figure 3B). Sequence of Lp01^p45kan^ demonstrated the presence of the specific point mutations and deletions we observed in Lp01 (Supplementary Table S2), confirming the identity of the strain to be Lp01 and differentiating it from the donor. Quantitative analysis of the regions associated with episomal and integrated p45 (Figure 3C) demonstrated that the number of p45 elements in both Phil-1 and Lp01^p45kan^ are comparable as shown by the *lvhB8* regions (p45) compared with *gyrB* (chromosome). The number of integrated copies of p45 are also comparable between Phil-1 and Lp01^p45kan^, demonstrating that the frequencies of site-specific recombination are similar. The only significant difference between Phil-1 and Lp01^p45kan^ is the number of episomal copies of p45 as measured by the difference in the frequency of *attP* between Phil-1 and Lp01^p45kan^ (*P* = .00047). This difference suggests that p45kan is maintained better in the episomal form than p45. Despite the presence of more episomal p45 in the Lp01^p45kan^ population than Phil-1, transcripts from the genes present on p45 are expressed at comparable levels (Figure 3D). These data suggest that p45kan will behave similarly to p45 after transfer to Lp01.

### p45 Restores Virulence to Lp01

**Figure 4.**
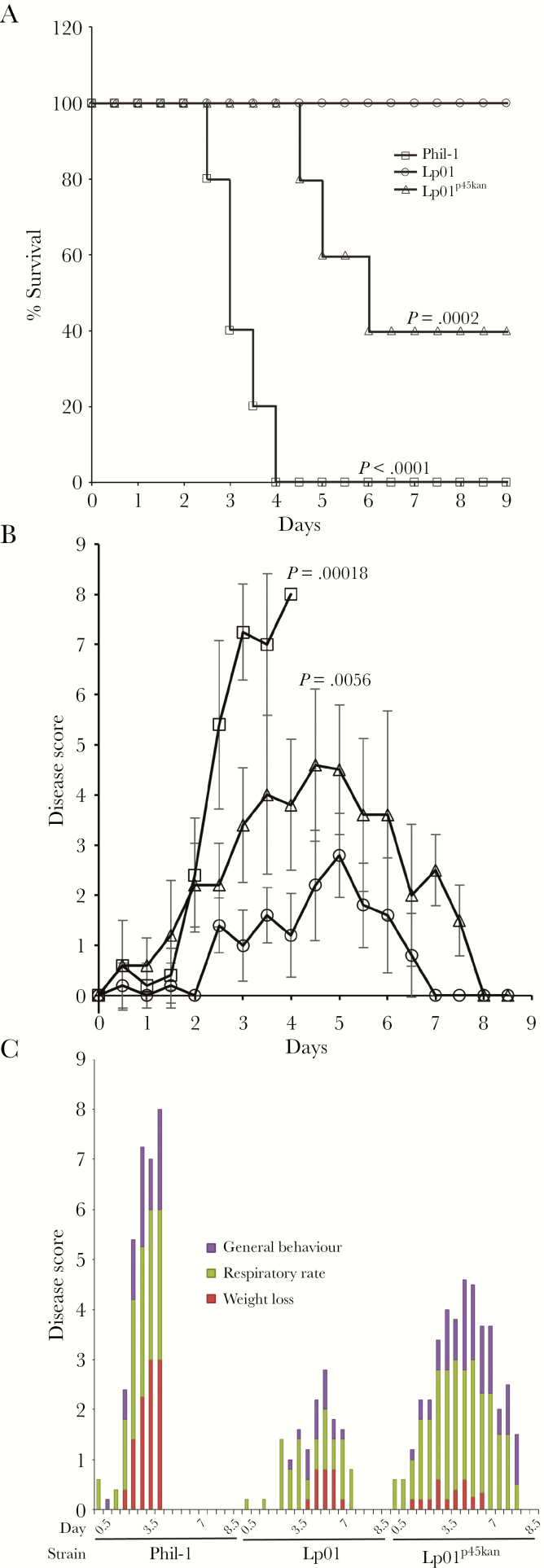
p45 plays a role in virulence for guinea pigs. Percentage of survival (A), disease scores (B), and breakdown of disease scores (C) for 5 guinea pigs per group infected by aerosol with *Legionella pneumophila* serogroup 1 Philadelphia wild-type strain (Phil-1), Lp01, or Lp01^p45kan^. Data are means and error bars are standard deviations for all animals surviving at each time point. Figure legend in A applies to A and B. *P* values shown are from the log-rank test (A) or analysis of variance (B). All statistical comparisons are between other strains and Lp01.

**Figure 5.**
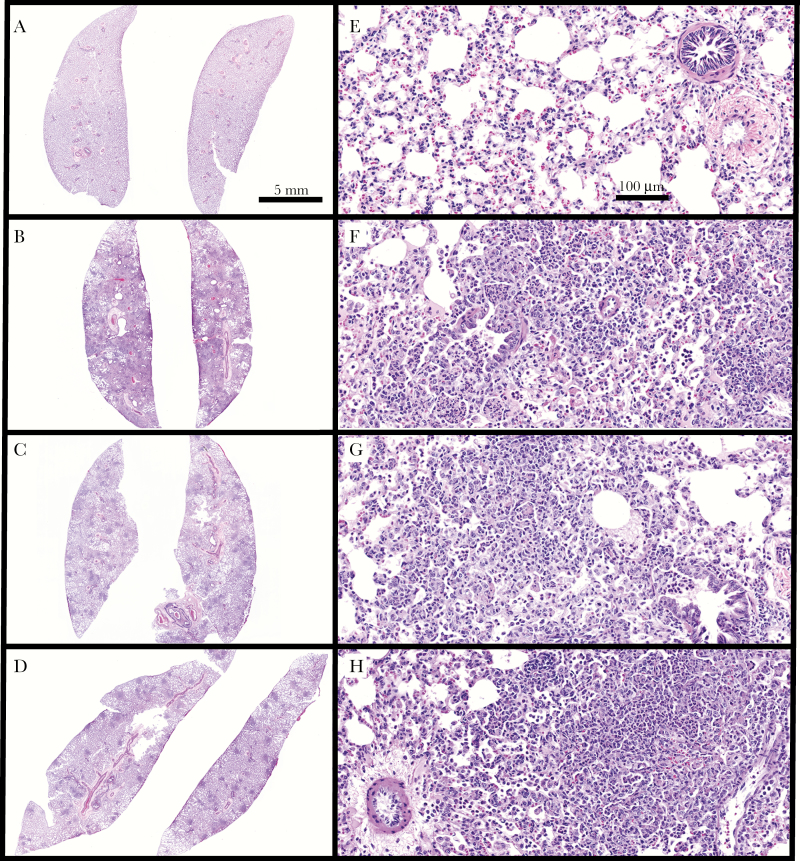
Lung pathology correlates with presence of p45. Hematoxylin and eosin stains of lungs from uninfected guinea pigs (A and E) or guinea pigs infected with *Legionella pneumophila* serogroup 1 Philadelphia wild-type strain ([Phil-1] B and F), Lp01 (C and G), or Lp01^p45kan^ (D and H) at low (A–D) or high (E–H) magnification, at 48 hours postinfection. Multifocal to coalescing inflammatory infiltrates fill the alveoli and occasional terminal bronchioles within all infected lungs (B–H) but are absent in uninfected lungs (A and E). Scale bar in A applies to A–D and scale bar in E applies to E–H.

We compared virulence of Phil-1, Lp01, and Lp01^p45kan^ in guinea pigs infected by aerosol (Figure 4). Animals infected with Phil-1 died within 4 days postinfection (Figure 4A) and exhibited severe disease within 2 days (Figure 4B and C). Animals infected with Lp01 displayed less severe disease, ultimately displaying no symptoms by day 7 postinfection (Figure 4B and C). Guinea pigs infected with Lp01^p45kan^ displayed similar disease symptoms to Phil-1 and mortality rates were higher than Lp01, with 3 of 5 animals succumbing to infection by 6 days postinfection (Figure 4A–C). Animals infected with Lp01^p45kan^ displayed more severe disease, particularly early loss of weight before 4 days, than those infected with Lp01 (Figure 4C). These observations suggest that the course of disease in animals infected with strains that carry p45 is more severe and more rapid than strains lacking p45. Histopathology on lungs at 48 hours postinfection with Phil-1, Lp01, and Lp01^p45kan^ was compared with uninfected animals (Figure 5). Macroscopically, the lungs from Phil-1-infected animals were markedly firm, wet, mottled tan to red, and consolidated with numerous pinpoint, firm foci. Animals infected by Lp01 had minimally to mildly firm and consolidated lungs with fewer, variably apparent, pinpoint, firm foci. Microscopically, animals infected with Phil-1 (Figure 5B and F) and Lp01^p45kan^ (Figure 5D and H) had moderate to severe coalescing bronchopneumonia characterized by numerous coalescing foci of infiltrating neutrophils, macrophages, and lymphocytes filling alveoli. Admixed with the inflammatory infiltrates was proteinaceous edema fluid, fibrin, karyorrhectic debris, and hemorrhage. Animals infected with Lp01 (Figure 5C and G) exhibited less pathological damage collectively compared with those infected with Phil-1. Guinea pigs infected with Lp01 displayed mild to moderate bronchiolitis with moderate alveolar pneumocyte accumulations progressing to acute/subacute stage bronchiolar and alveolar pneumonia, but these accumulations were not as abundant and less severe than those present in animals infected with Phil-1. Based on disease progress, mortality, and pathology, strain Lp01 is less virulent due to loss of the p45 element.

### *Legionella pneumophila* Containing p45 Induce Greater Interferon-γ

**Figure 6.**
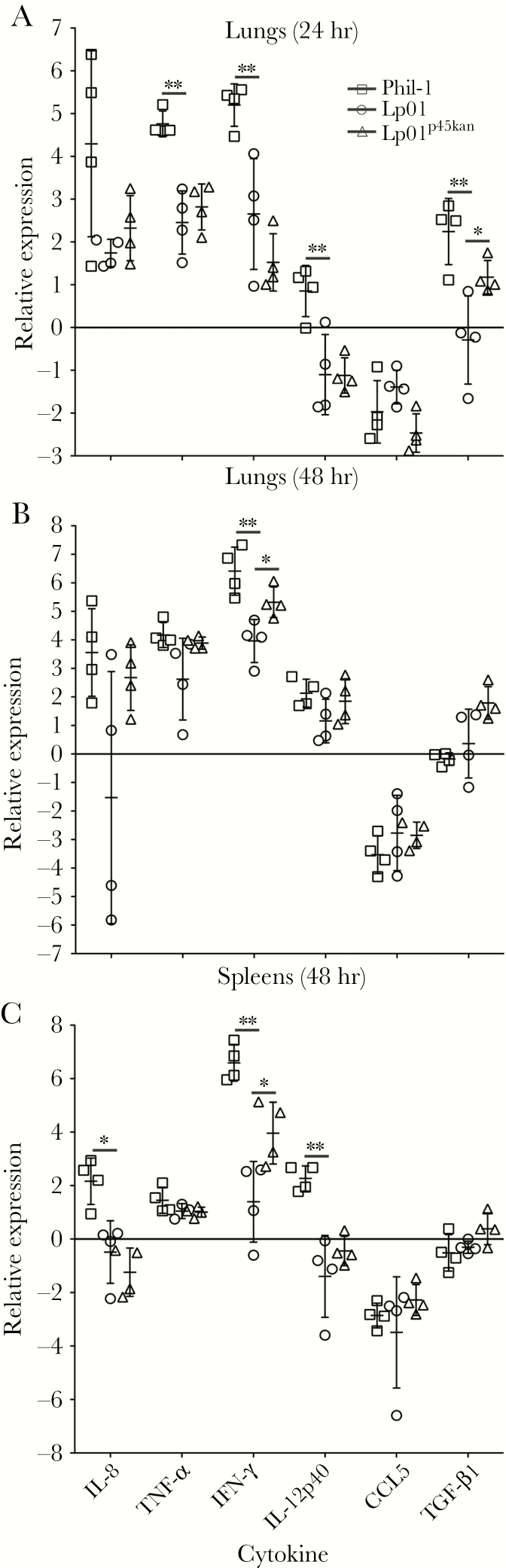
Immune response induced by p45 in guinea pigs. Cytokine expression was measured by quantitative reverse-transcriptase polymerase chain reaction on ribonucleic acid from lung homogenates at 24 (A) and 48 hours (B) and spleens at 48 hours (C) postinfection with *Legionella pneumophila* serogroup 1 Philadelphia wild-type strain (Phil-1), Lp01, and Lp01^p45kan^. Expression was calculated by the 2^−ΔΔC^T method relative to hypoxanthine guanine phosphoribosyltransferase transcripts from uninfected animals. Figure legend in A applies to A–C. Error bars represent standard deviations and data represent means using 4 animals per group. *, *P* < .05 and **, *P* < .01 compared with the Lp01 group of animals. IFN, interferon; IL, interleukin; TGF, transforming growth factor; TNF, tumor necrosis factor.

Cytokine transcript levels in the lungs 24 and 48 hours postinfection and spleens at 48 hours were compared. Expression of IL-8, IL-12p40, transforming growth factor (TGF)-β1, IFN-γ, CCL5, and TNF-α messenger ribonucleic acid (mRNA) was examined by quantitative reverse-transcriptase PCR (Figure 6). Guinea pigs infected with Phil-1 displayed higher expression of TNF-α, IFN-γ, IL-12p40, and TGF-β1 mRNA after 24 hours compared with Lp01 (Figure 6A). Tumor necrosis factor-β1 expression was significantly higher in lungs of animals infected with Lp01^p45kan^ at 24 hours compared with Lp01. It is interesting to note that, although the difference between Phil-1 and Lp01 in levels of IL-8 was not significant at 24 hours in lungs (*P* = .059), expression levels were highly variable and appeared bimodal. Comparing only the 3 high IL-8 animals infected with Phil-1 to those infected with Lp01 gives a *P* = .0028 by the Student’s *t* test, making it likely that IL-8 levels are often different, but the outbred nature of the animals can partially mask those differences. Interferon-γ expression in the lungs of animals infected with Phil-1 and Lp01^p45kan^ were higher than those infected with Lp01 at 48 hours postinfection (Figure 6B), suggesting that p45 plays a role in a sustained IFN-γ host response. Once again, IL-8 levels were highly variable, but this time particularly in Lp01, where if the lower 3 animals are compared with Phil-1 and Lp01^p45kan^ the *P* = .017 and *P* = .024, respectively. Spleens of guinea pigs infected with Phil-1 at 48 hours after infection display higher expression of IL-8, IL-12p40, and IFN-γ compared with those infected by Lp01 (Figure 6C). Similar to the lungs at the same time point and Phil-1, spleens of animals infected with Lp01^p45kan^ also expressed higher levels of IFN-γ relative to Lp01. These data suggest that p45 is involved in host mortality through overinduction of a robust and sustained proinflammatory response involving primarily IFN-γ but also IL-12, TNF-α, and IL-8.

## Discussion

**Figure 7.**
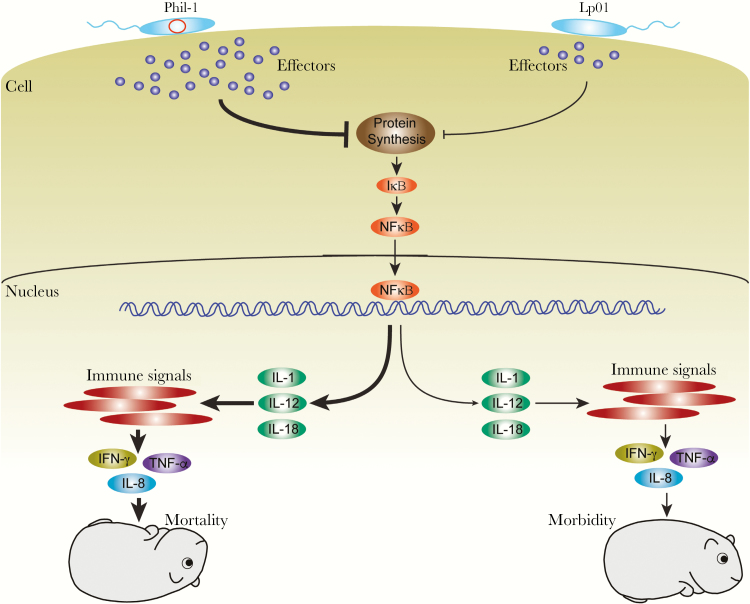
Mechanism leading to p45-related guinea pig mortality. Based on previously published evidence and data from the current study, abundant production and secretion of effectors mediated by the presence of 2 type IV secretion systems (p45 *lvh* and *dot/icm*) in *Legionella pneumophila* serogroup 1 Philadelphia wild-type strain (Phil-1) leads to enhanced translation inhibition that reduces IκΒ, allowing sustained activation of nuclear factor (NF)-κΒ and increased transcription and secretion of proinflammatory cytokines interleukin (IL)-1, IL-12, and IL-18 by infected cells. Sustained production of IL-1, IL-12, and IL-18 increase proinflammatory interferon (IFN)-γ, tumor necrosis factor (TNF)-α, and IL-8 production leading to multiorgan failure and death of guinea pigs. In contrast, although the same pathways are induced by Lp01, the absence of sustained IL-12, and most likely IL-1 and IL-18, production that would normally be induced by effector production and section, the proinflammatory response can be controlled sufficiently to allow dampening of the response by transforming growth factor-β and recovery of the guinea pigs.

We found that the p45 element is associated with virulence and impacts mortality due to overinduction of a proinflammatory response. Effectors secreted by type IV secretion systems in *L pneumophil*a play an important role in the inflammatory response [4–7, 35–37], and the *lvh/lvr* locus that is located on p45 can impact effector secretion [38, 39]. We offer evidence that p45-mediated overinduction of the inflammatory response is involved in mortality. Because *the* Lvh type IVA secretion system on p45 is required for optimal secretion of SdeA [39], a SidE orthologue toxic to eukaryotic cells through inhibition of translation [40, 41], the effects of p45 on cytokine levels are most likely due to inhibition of protein synthesis (Figure 7). There is a good deal of evidence that blockage of protein synthesis by *L pneumophila* leads to IκΒ degradation and, thereby, activation of NF-κβ [6, 7, 42] as well as induction of IL-1 [4, 5, 35]. Activation of NF-κβ and IL-1 pathways lead to an inflammatory response that involves IL-1, IL-12, IL-18, IFN-γ, TNF-α, and IL-8 [5, 7, 9, 35]. Our data support these earlier observations, because induction of IL-12, IFN-γ, TNF-α, and IL-8 correlates with p45, suggesting a similar mechanism of mortality through greater effector secretion inhibiting protein synthesis. The impact of p45 on disease explains observations that *Legionella* strains differ in their ability to cause severe disease and epidemics [13, 21, 29–32]. This information could be used, in combination with other loci that correlate with more virulent strains such as *rtxA* [13, 43, 44], to identify strains that have the potential to cause disease and differentiate them from the larger number of less virulent environmental strains [11–13]. A proinflammatory response develops in response to *L pneumophila* infection, based on increased expression of TNF-α, IL-1β, and IL-12 and from human tissues, IL-8 [7]. Our data align with previous reports, while emphasizing the importance of IFN-γ when p45 is present. Overinduction of type II IFN is likely a contributing factor in the mechanisms of Legionnaires’ disease mortality, revealing an important role for p45 in virulence of *L pneumophila*, supported by prevalence of the genes carried by p45 in clinically relevant strains [13, 29]. The important role of IFN-γ points toward potential strategies for patient treatment by specifically blocking overinduction.

The guinea pig model has been useful in study of Legionnaires’ disease [3] and can sensitively measure differences in virulence [45]. It is interesting to note that the 50% infectious dose for *L pneumophila* in guinea pigs is 129 organisms, yet the LD_50_ (lethal dose, 50%) is 1.4 × 10^5^ [46]. These observations are consistent with an epidemiological picture where many people become infected subclinically, but sporadically moderate to large epidemics and mortalities are observed [47]. The strains Phil-1 and 130b or Wadsworth, the parent strain of AA100, were responsible for epidemics with mortalities [3, 34], consistent with our observation of high mortality rates in guinea pigs. Strains Phil-1, AA100, and JR32 led to pathology, mortality, and higher bacterial loads; however, Lp01 appears to be controlled, and the animals recover at 7 days postinfection. These observations support an important role for p45 in Legionnaires’ disease and provide a valuable system for analysis of virulence factors that impact the severity of Legionnaires’ disease. Transfer of the p45 element into strains with virulence gene mutations already constructed could allow the importance of each locus in severity of disease to be evaluated. Furthermore, construction of a series of mutations in p45 could allow elucidation of the specific factors involved in the severity of Legionnaires’ disease, their degree of importance in disease, and the molecular mechanisms by which they function. In particular, analysis of the more than 300 effectors identified in this system could allow elucidation of a subset that are critical for pathogenesis, providing additional targets to prevent severe disease due to this relatively ubiquitous organism as well as for screening water systems to identify those that represent a threat to public health for targeted mitigation.

Although transfer of p45 into Lp01 recovered the majority of virulence, Lp01^p45kan^ did not appear to be as virulent as Phil-1. There are 3 possible reasons that p45kan may not fully complement Lp01: (1) presence of greater episomal copies in Lp01^p45kan^ leading to suboptimal virulence gene regulation, (2) presence of kanamycin resistance creates a metabolic load reducing virulence, or (3) mutations in Lp01 other than p45 loss are responsible for the remaining difference. Because p45 carries the noncoding RNA lpr0035, located within the p45 integration site, and loss of lpr0035 reduces entry and intracellular multiplication in amoeba and macrophages, mobility of p45 could impact expression of this noncoding RNA [48]. Work is ongoing to test the effects of these potential differences, but, in the meantime, p45kan allows analysis of the role of individual genes on p45 in virulence. In addition, analysis of the distribution of p45 and similar mobile elements that may be associated with virulence is critical to better understand strains that cause Legionnaires’ disease.

## Conclusions

Although studies have linked mobile genetic elements to *Legionella* virulence-related characteristics [49, 50], few have evaluated their implications in pathogenesis. The MGEs, mobile integrative elements (MIE), and integrative conjugative elements (ICE) are common in *Legionella* spp [24]. The p45 element shares characteristics with MIEs, including higher G+C content, flanking tRNA sequences [48], and an integrase [26]. In addition, p45 encodes conjugation machinery, phage-related genes, and a type IVa secretion system, suggesting that p45 should be classified as an ICE [26]. Because p45 can be conjugated from Phil-1 to Lp01, we propose that p45 be considered an ICE. The most likely genes responsible for pathogenesis are those present within the Lvh type IVA secretion system because of its known role in secretion of effectors that impact protein synthesis and the inflammatory response. However, there are likely other virulence genes on p45, making continued analyses important. Prior studies support a regulatory role for genes on p45 in virulence and during disease [13, 27, 39, 43]. *Legionella pneumophila* Thunder Bay and HL06041035, members of SG6 and SG12, respectively, have been implicated in disease and carry predicted MIEs encoding *lvh/lvr* [29], the same type IVA secretion system as in Phil-1. It is interesting to note that *Legionella longbeachae* strain D-4968 carries a mobile element with the *lvh/lvr* region, but *L longbeachae* strain NSW-150 lacks these loci [29], serendipitously representing 2 strains that might allow analysis of the role of the *lvh* type IVA secretion system in severity of *L longbeachae* disease. Although additional studies are needed to better understand how *lvh* type IVA secretion systems and possibly other components on p45 impact *Legionella* disease severity, our data demonstrate that the p45 ICE plays an important role in *Legionella* pathogenesis through increasing IFN-γ expression after infection.

## Supplementary Data

Supplementary materials are available at *The Journal of Infectious Diseases* online. Consisting of data provided by the authors to benefit the reader, the posted materials are not copyedited and are the sole responsibility of the authors, so questions or comments should be addressed to the corresponding author.

jiz340_suppl_Supplementary_Information

## Notes

***Acknowledgments*.** We thank Ralph Isberg, Michele Swanson, and Howard Shuman for providing bacterial strains. We recognize the technical and theoretical contributions made to this project by the late Suat L. G. Cirillo. We thank David McMurray and Samantha Bell for their input, suggestions, and editing.

***Financial support.*** This work was funded by National Institutes of Health Grant AI40165 (to J. D. C.).

***Potential conflicts of interest.*** All authors: No reported conflicts of interest. All authors have submitted the ICMJE Form for Disclosure of Potential Conflicts of Interest.
