# Supplementary material for: Legionnaires’ Disease Mortality in Guinea Pigs Involves the p45 Mobile Genomic Element
Source: J Infect Dis. 2019 Jul 2;220(10):1700–10. doi: 10.1093/infdis/jiz340 (PMC6782102; doi:10.1093/infdis/jiz340)
Supplement: jiz340_suppl_Supplementary_Information [file jiz340_suppl_supplementary_information.docx]

**Supporting Information**

**Supplementary Materials and Methods**

**Bacterial strains and growth conditions.** Phil-1 and its derivative strain, Lp01, were provided by Ralph Isberg and Michele Swanson. Lp01 is streptomycin resistant and more easily transformed than Phil-1 [1]. JR32, a gift from Howard Shuman, is also derived from the wild type parent strain Phil-1, and is similarly streptomycin resistant and easily transformed [2]. AA100 was isolated as a streptomycin resistant mutant of 130b [3], a clinical isolate from the Wadsworth Veterans Administration Hospital in Los Angeles, CA [4]. The *Legionella* strains were grown as described previously [5], on buffered charcoal yeast extract (BCYE) agar plates or shaken in buffered yeast extract (BYE) broth at 37°C. *L. pneumophila* used for experiments were grown from 4°C stock lawns grown from -80°C glycerol stocks. Stock lawns were kept and used at 4°C for no longer than 5 days and passaged no more than twice, as described previously [6]. *Escherichia coli* XL1-Blue was grown in Luria-Bertani (LB) liquid medium or agar plates at 37°C.

**Confirmation of chromosomal integration and presence of p45.** Genomic DNA from *L. pneumophila* Phil-1 and Lp01 cultures was isolated by standard phenol/chloroform extraction as previously described [6]. Quantitative PCR was performed using SYBR Green PCR Master Mix (Applied Biosystems), ~50 ng/μl of genomic DNA, and oligonucleotides targeting *attB, attP*, the *attP/attB* junction, *lpg1249*, and *gyrB/lpg0004* described in Table S1. Quantities were determined by comparing target C_T_ values from *L. pneumophila* Phil-1 with those of *gyrB* and then with the same calculated values from Lp01 DNA, which lacks the p45 MIE using the 2^-ΔΔC^T method [7]. Samples were run in triplicate and each primer set was accompanied by negative control wells with no template.

**Vector construction.** The plasmid pMV262 containing the *aph* kanamycin resistance cassette was isolated from XL1-Blue *E. coli* strain Ψec485 as previously described [8], and digested with restriction enzymes, *AfeI* and *BamHI*-HF. The digested pMV262 was then ligated with a ~200 bp fragment amplified by PCR from the p45 MIE of *L. pneumophila* Phil-1, also digested by *BamHI*-HF and *AfeI*. The ligated product was transformed by electroporation into *E. coli* XL1-Blue as described previously [9] and selected on BCYE with kanamycin (25 μg/ml). The resulting plasmid, pJDC321, in *E. coli* Ψec551 was confirmed to contain the *aph* cassette by PCR and restriction enzyme digestion, and transformed into *L. pneumophila* Phil-1 by electroporation. Bacteria successfully transformed by pJDC321 were selected using BCYE with kanamycin (25 μg/ml). Colonies were verified to contain pJDC321 and its integration within the p45 element by homologous recombination, using PCR. The resulting strain, Phil-1^p45kan^, with a p45 element carrying kanamycin resistance, was used as the donor strain for conjugation of p45.

**Conjugation of p45.** *Legionella* cultures grown as lawns on BCYE agar plates at 30°C and 5% CO_2_ for 2-3 days, were used to inoculate BYE liquid broth, and grown to post-exponential phase (optical density at 600 nm (OD_600_) = 1.2 - 1.4) with shaking at 30°C. The bacteria were then diluted to an OD_600_ = ~1.0. The donor strain, Phil-1^p45kan^, carrying kanamycin resistance inserted within the p45 element via homologous recombination. The recipient strain, Lp01, that carries streptomycin resistance, was selected against the Phil-1 background donor strain using streptomycin. Cultures of the donor, the recipient, and a 1:1 ratio of both were mixed in the presence of DNase (1 µg/µl) and placed on sterile filter discs with 0.45 μm pores using vacuum filtration as described previously [10, 11]. The discs were then placed with the bacterial side up on BCYE plates at 30°C in the presence of 5% CO_2_ for 4 hours. The bacteria were then suspended in BYE, diluted, and plated to select for Lp01 carrying p45kan (Lp01^p45kan^) trans-conjugates on BCYE containing kanamycin (25 μg/ml) and streptomycin (200 μg/ml). The efficiency of conjugation was determined by dividing the number of colony forming units (CFU) that grew on the selective BCYE containing streptomycin (200 μg/ml) and kanamycin (25 μg/ml), with the number that grew on non-selective BCYE plates.

**Confirmation of Lp01^p45kan^.** Oligonucleotides listed in Table S1 were designed to verify the presence of p45, its integration into the chromosome, and to distinguish the Lp01 background from that of Phil-1. The presence of p45 and its site-specific integration into the chromosome were determined by targeting p45-encoded *lvhB8*, disruption of the chromosomal *attB* site, and the *attP/attB* p45-chromosomal integration junctions. In order to screen for the Lp01 genetic background by PCR, oligonucleotides were designed to detect a single nucleotide polymorphism within *rpsL* (*lpg0234*) [12]. The 3’ tip of the forward primer was designed to complement the guanine residue of *rpsL* in Lp01, rather than the adenine encoded by Phil-1. Genomic DNA (~80 μg) was isolated as described previously [6] from colonies selected on BCYE containing kanamycin (25 μg/ml) and streptomycin (200 μg/ml) resulting from Phil-1^p45kan^/Lp01 conjugation. PCR was carried out with an Applied Biosystems Veriti thermocycler, products were separated by gel electrophoresis, and visualized using ethidium bromide staining.

**Transcript analysis in *Legionella*.** RNA was isolated from *L. pneumophila* Phil-1, Lp01, and Lp01^p45kan^ cultures suspended in TRIZOL, as we described previously [13]. The Super Script III Reverse Transcription System from Invitrogen was used to convert RNA samples to cDNA following manufacturer’s recommendations. Approximately 50 ng/μl cDNA per reaction was used for RT-qPCR with primers which target transcripts from *lpg1266, lpg1254, lpg1241, lpg1229, lpg1249*, and *gyrB/lpg0004* (Table S1), and SYBR Green PCR Master Mix by Applied Biosystems following manufacturer’s recommendations. Experiments were done with a Thermofisher Scientific StepOnePlus Real Time PCR System, in 96-well plates. Each reaction target was accompanied by negative control wells with no template, and samples were run in triplicate. Expression values were calculated by comparing target C_T_ values to those of *gyrB.* Those values (ΔC_T_) were then compared to Lp01 using the 2^-ΔΔC^T method [7].

Sequencing was performed by Eton Biosciences using genomic DNA (~80 μg) isolated from *L. pneumophila* Phil-1, Lp01, and Lp01p45kan of genetic regions which harbor small polymorphism differences between the published genomes of Phil-1 (AE017354.1) [14] and Lp01 [12] located in *lpg0324, lpg0671, lpg0716, lpg0718, lpg2506*, and *lpg2669* gene regions (Table S2). Oligonucleotides used for PCR amplification, RT-qPCR, and sequencing are shown in Table S1.

**Sequencing of *Legionella* genomic regions.** Sequencing was performed by Eton Biosciences using genomic DNA (~80 μg) isolated from *L. pneumophila* Phil-1, Lp01, and Lp01p45kan of genetic regions which harbor small polymorphism differences between the published genomes of Phil-1 (AE017354.1) [14] and Lp01 [12] located in *lpg0324, lpg0671, lpg0716, lpg0718, lpg2506*, and *lpg2669* gene regions (Table S2). Oligonucleotides used for PCR amplification, RT-qPCR, and sequencing are shown in Table S1.

**Guinea pig infections**. Female Hartley guinea pigs (250 - 300 g) obtained from Charles River Laboratories were infected with *Legionella pneumophila* cultures, grown on BCYE plates at 37°C and 5% CO_2_ and suspended in sterile ddH_2_O, using a Madison aerosol chamber to give an infectious dose of ~5 x 10^5^ cfu/lungs with each strain [3, 15]. The bacterial cultures normalized using OD_600_ to achieve infection with similar numbers of bacteria within the lungs. Animals infected with *L. pneumophila* were monitored and scored for disease severity using three criteria: 1) weight loss, 2) respiratory rate, and 3) general behavior that included lethargy, skin coloration, and fur ruffling. When necessary, due to severe disease, humane euthanasia was carried by overdose of pentobarbital (FatalPlus). At necropsy, upper right lobe of the lungs and half of the spleen were collected in 5 ml sterile ddH_2_O, homogenized, diluted, and plated on BCYE agar containing 5 μg/ml polymixin B, and incubated in 37°C and 5% CO_2_ for 3-6 days to determine CFU present. Slices of the remaining lungs and spleens were either immediately added to, homogenized, and frozen at -80°C in TRIZOL reagent for RNA or fixed in 10 ml buffered formalin at 22°C (RT) for later embedding and staining for histopathology. Survival experiments were conducted with five guinea pigs per infection group, while the immune response experiments utilized four guinea pigs per time point per infection group.

**Cytokine expression analyses.** RNA was isolated from tissue samples frozen in TRIZOL, as we described previously [13]. The Super Script III Reverse Transcription System from Invitrogen was used to make cDNA following the manufacturer’s recommendations. Approximately 50 ng/μl cDNA per reaction was used for qRT-PCR with primers that target transcripts for guinea pig IL-8, IL-12p40, TNF-α, IFN-γ, TGF-β1, CCL5, and HPRT (Table S1) [16-18] using SYBR Green PCR Master Mix from Applied Biosystems. Experiments were carried out using the Thermofisher Scientific StepOnePlus Real Time PCR System in 96-well plates. Expression was calculated by comparing sample target C_T_ values to those of hypoxanthine-guanine phosphoribosyltransferase (HPRT) and uninfected guinea pigs using the 2^-ΔΔC^T method [7]. Samples were run in triplicate, and each primer set was accompanied by negative controls lacking cDNA template.

**Histopathology of guinea pig lung tissue.** All animals necropsied after euthanasia 24 or 48 hours following *L. pneumophila* infection were analyzed for histopathology. Sections of lung, liver, spleen and kidney were collected for microscopic evaluation. Tissues were trimmed, processed routinely, embedded in paraffin, and stained with hematoxylin and eosin, as we describe previously [3]. Microscopic evaluation was conducted by two board-certified veterinary pathologists in a blinded fashion.

**Supplementary Tables**

**Table S1.** Oligonucleotides used.

|  | **Primer Name**  **(RE cut site)** | **Nucleotide Sequence 5'-3'** | **Target Gene Annotated ID^a^** | **Target**  **Gene/Region** | **Target Organism** | **Product Size (bp)** |
| --- | --- | --- | --- | --- | --- | --- |
| **Cloning** |  |  |  |  |  |  |
|  | P45inst-F (*AfeI*) | ATATAGCGCTAGGAGCTAAATGTGCAGC | *lpg1237* | p45 | *L. pneumophila* | 206 |
|  | P45inst-R (*BamHI*) | ATATGGATCCTTTGCTCCCGAAAGCCTG |  |  |  |  |
| **PCR** |  |  |  |  |  |  |
|  | P45t2RE-F1 (*BamHI*) | TATAGGATCCTCCAAGCCAGAAAAATG | *lpg1237* | p45 | *L. pneumophila* | 200 |
|  | P45t2RE-R1 (*AatII*) | TATAGACGTCTGATGGAGTGAAGGGTTG |  |  |  |  |
|  | P45-262F1 | CGTCGACATCGATAAGCTTCG |  | p45kan | *L. pneumophila* | 300 |
|  | P45-262R1 | GGCAGGTGTGTTATTGCTATGC |  |  |  |  |
|  | attPF^b^ | AAGGCAATCCTGTTCGCATAC |  | p45 | *L. pneumophila* | 600 |
|  | attPR | TCTCCAAGCCACCAATAAAGC |  |  |  |  |
|  | LppF^c^ | TATATTGATGGCGGTGCTGCC |  | *attB* | *L. pneumophila* | 550 |
|  | LppR | ATCGAGATTGAGTTTACATCCC |  |  |  |  |
|  | Lp01rpsL-F ^d^ | GCACACCAGGTAAATCCC | *lpg0324* | *rpsL* | *L. pneumophila* | 573 |
|  | Lp01rpsL-R | AACGTGATGGTAGGTCGC |  |  |  |  |
| **qPCR** |  |  |  |  |  |  |
|  | qP45-F | TGATGCATCAAGCTATCGCG |  |  | *L. pneumophila* | 200 |
|  | qP45-R | ATACCACTTCTGCCAATGCG |  |  |  |  |
|  | qCRM-F | AAGGTATCGGAAACCGAACC |  |  | *L. pneumophila* | 200 |
|  | qCRM-R | CTTCCTGGATAAAGAGGAGC |  |  |  |  |
| **Sequencing** |  |  |  |  |  |  |
|  | rpsLMut-F | TCCATAACAGACAGCGCACC | *lpg0324* | *rpsL* | *L. pneumophila* | 466 |
|  | rpsLMut-R | AGACGCGGTGTATGTACTCG |  |  |  |  |
|  | rpsL-N-F | CGTTGATGAACTTAGCAAGCAGTTC |  |  |  |  |
|  | Phil-ndh-F3 (*AatII*) | ATATGACGTCAAGCACCGCCCTGTTTTGC | *lpg0671* | *ndh* | *L. pneumophila* | 976 |
|  | Phil-ndh-R3 (*NheI*) | ATATGCTAGCTTTAGCCTTGATGGTTTGCC |  |  |  |  |
|  | ndh-N-F | TCACACCATCGCTATCAATTTGC |  |  |  |  |
|  | hpMut-F | GATACTGGCGGATGGTTTGG | *lpg0716* | *h.p.*^e^ | *L. pneumophila* | 510 |
|  | hpMut-R | CAGTGGCAGTACCACTTTCG |  |  |  |  |
|  | hp-N-F | CATCACTCTCAACTTCAGCAGAG |  |  |  |  |
|  | gspMut-F | CTGCCAACTTAATGGCAACC | *lpg0718* | *gsp* | *L. pneumophila* | 337 |
|  | gspMut-R | ACCATTGTCGCTATGGGATC |  |  |  |  |
|  | gsp-N-F | ACCAAATCCATGCGCATCCC |  |  |  |  |
|  | luxNMut-F | CTATCTCGTGCAAACAAGGC | *lpg2506* | *luxN* | *L. pneumophila* | 498 |
|  | luxNMut-R | AACTGAAGATGCAAGCCACC |  |  |  |  |
|  | luxN-N-F | GGCACCAATGATTATTGCTGC |  |  |  |  |
|  | ftsEMut-F | CACCAATAGATGCTGAGGCG | *lpg2669* | *ftsE* | *L. pneumophila* | 641 |
|  | ftsEMut-R | AAGTAACCCATGGCCAGCAC |  |  |  |  |
|  | ftsE-N-F | GGAAGAGTCCCCAGAGGAGC |  |  |  |  |
| **RT-qPCR** |  |  |  |  |  |  |
|  | qLvhB8-F | TGATGCATCAAGCTATCGCG | *lpg1249* | *lvhB8* | *L. pneumophila* | ~200 |
|  | qLvhB8-R | ATACCACTTCTGCCAATGCG |  |  |  |  |
|  | qAttB-F | GCTATAAGGGTAGACTTGCG |  | *attB* | *L. pneumophila* | ~200 |
|  | qAttB-R | TAAAGCAAGGGTGCTTTAGG |  |  |  |  |
|  | qAttP-F | TTGCATAGAGACACATGGAG |  | *attP* | *L. pneumophila* | ~200 |
|  | qAttP-R | TTTGCACCCTGAGTGCAC |  |  |  |  |
|  | qGyrB-F | AGCATCATCACCTGTTGGAG | *lpg0004* | *gyrB* | *L. pneumophila* | ~200 |
|  | qGyrB-R | CCATGCAATGGAATGATGGC |  |  |  |  |
|  | qMEase-F | AAGCGAATAGATGCCTACGC | *lpg1266* | methylase | *L. pneumophila* | ~200 |
|  | qMEase-R | AGGGAGTATGATGGCATTGC |  |  |  |  |
|  | qLvhB4-F | TGGAATTGCCAGTGAAACCC | *lpg1254* | *lvhB4* | *L. pneumophila* | ~200 |
|  | qLvhB4-R | GAGGTAATCAGAGAGGCTCG |  |  |  |  |
|  | qTraA-F | GCACCACTTTACTACCTGCC | *lpg1241* | *traA* | *L. pneumophila* | ~200 |
|  | qTraA-R | TATCCAAGCAGGGATGGAGG |  |  |  |  |
|  | qSSR-F | ATGCCGTAAATCATGCCAGC | *lpg1229* | recombinase | *L. pneumophila* | ~200 |
|  | qSSR-R | AGCAGTTCGCGCTATATACC |  |  |  |  |
|  | TGFb1-GP-F | CATCGATATGGAGCTGGTGAAG |  | TGF-β1 | *Cavia porcellus* | ~200 |
|  | TGFb1-GP-R | GCCGTAATTTGGACAGGATCTG |  |  |  |  |
|  | TNFa-GP-F | CCTACCTGCTTCTCACCCATACC |  | TNF-⍺ | *Cavia porcellus* | ~200 |
|  | TNFa-GP-R | TTGATGGCAGAGAGAAGGTTGA |  |  |  |  |
|  | IFNg-GP-F | ATTTCGGTCAATGACGAGCAT |  | IFN-𝜸 | *Cavia porcellus* | ~200 |
|  | IFNg-GP-R | GTTTCCTCTGGTTCGGTGACA |  |  |  |  |
|  | CCL5-GP-F | CTGGCCCACTGCTTAGCAAT |  | CCL5 | *Cavia porcellus* | ~200 |
|  | CCL5-GP-R | CCTTGCTTCTTTGCCTTGAAA |  |  |  |  |
|  | IL12p40-GP-F | CCACAGTTTCATGCCACAAGA |  | IL-12p40 | *Cavia porcellus* | ~200 |
|  | IL12p40-GP-R | CCATTCGCTCCACGATGAG |  |  |  |  |
|  | IL8-GP-F2 | TAGGGTGGCAGATTTAACTCA |  | IL-8 | *Cavia porcellus* | 121 |
|  | IL8-GP-R2 | TCAGGAATTGGCTTGCTAC |  |  |  |  |
|  | HPRT-GP-F | AGGTGTTTATCCCTCATGGACTAATT |  | HPRT | *Cavia porcellus* | ~200 |
|  | HPRT-GP-R | CCTCCCATCTCCTTCATCACAT |  |  |  |  |

^a^Annotations and sequence obtained from the National Center for Biotechnology Information accession number AE017354.1[14].

^b^Used with LppR to detect the *attP/attB* junction on the integrated p45 element within the chromosome.

^c^Used with attPR to detect the *attB/attP* junction on the integrated p45 element within the chromosome.

^d^Differentiates G nucleotide in Lp01 from A nucleotide in Phil-1 within the *rpsL* gene that confers streptomycin resistance to Lp01[12].

^e^h.p. = hypothetical protein.

| **Annotated ID^a^** | | **Gene** | **Sequence Present in Strain** | | | | |
| --- | --- | --- | --- | --- | --- | --- | --- |
|  |  |  | **Phil-1^a^** | **Phil-1^b^** | **Lp01^c^** | **Lp01^b^** | **Lp01^p45kan b^** |
| *lpg0324* | *rpsL* | | A | A | G | G | G |
| *lpg0671* | *ndh* | | GGCCGAAAT^d^ | GGCCGAAAT^d^ | 9 bp Del^d^ | 9 bp Del^d^ | 9 bp Del^d^ |
| *lpg0716* | hypothet^e^ | | C | C | T | C | C |
| *lpg0718* | proton sym^e^ | | C | C | T | C | C |
| *lpg2506* | *luxN* | | C | C | A | A | A |
| *lpg2669* | *ftsE* | | T | T | C | T | T |

**Table S2.** Validation of strains by sequencing.

^a^Annotations and sequence from *Legionella pneumophila* serogroup 1 strain Philadelphia (Phil-1) National Center for Biotechnology Information accession number AE017354.1 [14].

^b^Sequence from the current study for *Legionella pneumophila* serogroup 1 strains Phil-1, Lp01 or Lp01^p45kan^.

^c^Sequence from *Legionella pneumophila* strain Lp01 previously published [12].

^d^Sequence present in Phil-1 and deleted (Del) in all Lp01 derivatives.

^e^Hypothet. = hypothetical protein, proton sym = proton symporter.

**Supplementary References**

1. Berger KH, Isberg RR. Two distinct defects in intracellular growth complemented by a single genetic locus in *Legionella pneumophila*. Mol Microbiol **1993**; 7:7-19.

2. Wiater LA, Sadosky AB, Shuman HA. Mutagenesis of Legionella pneumophila using Tn903 dlllacZ: identification of a growth-phase-regulated pigmentation gene. Mol Microbiol **1994**; 11:641-53.

3. Moffat JF, Edelstein PH, Regula DP, Jr., Cirillo JD, Tompkins LS. Effects of an isogenic Zn-metalloprotease-deficient mutant of *Legionella pneumophila* in a guinea-pig model. Molec Microbiol **1994**; 12:693-705.

4. Edelstein PH, Nakahama C, Tobin JO, et al. Paleoepidemiologic investigation of Legionnaires disease at Wadsworth Veterans Administration Hospital by using three typing methods for comparison of legionellae from clinical and environmental sources. Journal of Clinical Microbiology **1986**; 23:1121-6.

5. Edelstein PH. Improved semiselective medium for isolation of *Legionella pneumophila* from contaminated clinical and environmental specimens. J Clin Microbiol **1981**; 14:298-303.

6. Cirillo SLG, Lum J, Cirillo JD. Identification of novel loci involved in entry by Legionella pneumophila. Microbiology **2000**; 146:1345-59.

7. Livak KJ, Schmittgen TD. Analysis of relative gene expression data using real-time quantitative PCR and the 2(-Delta Delta C(T)) Method. Methods **2001**; 25:402-8.

8. El-Etr SH, Subbian S, Cirillo SLG, Cirillo JD. Identification of Two Mycobacterium marinum Loci That Affect Interactions with Macrophages. Infection and Immunity **2004**; 72:6902-13.

9. Dower WJ, Miller JF, Ragsdale CW. High efficiency transformation of E. coli by high voltage electroporation. Nucleic Acids Research **1988**; 16:6127-45.

10. Dreyfus LA, Iglewski BH. Conjugation-mediated genetic exchange in Legionella pneumophila. Journal of Bacteriology **1985**; 161:80.

11. Lautner M, Schunder E, Herrmann V, Heuner K. Regulation, Integrase-Dependent Excision, and Horizontal Transfer of Genomic Islands in Legionella pneumophila. Journal of Bacteriology **2013**; 195:1583-97.

12. Rao C, Benhabib H, Ensminger AW. Phylogenetic Reconstruction of the Legionella pneumophila Philadelphia-1 Laboratory Strains through Comparative Genomics. PLOS ONE **2013**; 8:e64129.

13. Park B, Subbian S, El-Etr SH, Cirillo SL, Cirillo JD. Use of gene dosage effects for a whole-genome screen to identify Mycobacterium marinum macrophage infection loci. Infect Immun **2008**; 76:3100-15.

14. Chien M, Morozova I, Shi S, et al. The Genomic Sequence of the Accidental Pathogen Legionella pneumophila. Science **2004**; 305:1966-8.

15. Berendt RF, Young HW, Allen RG, Knutsen GL. Dose-Response of Guinea Pigs Experimentally Infected with Aerosols of Legionella pneumophila. The Journal of Infectious Diseases **1980**; 141:186-92.

16. Allen SS, McMurray DN. Coordinate Cytokine Gene Expression In Vivo following Induction of Tuberculous Pleurisy in Guinea Pigs. Infection and Immunity **2003**; 71:4271-7.

17. Cho H, Lasco TM, Allen SS, Yoshimura T, McMurray DN. Recombinant Guinea Pig Tumor Necrosis Factor Alpha Stimulates the Expression of Interleukin-12 and the Inhibition of Mycobacterium tuberculosis Growth in Macrophages. Infection and Immunity **2005**; 73:1367-76.

18. Yamada H, Udagawa T, Mizuno S, Hiramatsu K, Sugawara I. Newly Designed Primer Sets Available for Evaluating Various Cytokines and iNOS mRNA Expression in Guinea Pig Lung Tissues by RT-PCR. Experimental Animals **2005**; 54:163-72.
